# 234 Unraveling the Link: How Emotional Self-Regulation and Procrastination Affect Physical Activity

**DOI:** 10.1093/eurpub/ckae114.118

**Published:** 2024-09-26

**Authors:** Radoslawa Herzog-Krzywoszanska, Lukasz Krzywoszanski, Wiktor Potoczny

**Affiliations:** University of the National Education Commission, Krakow, Poland; University of the National Education Commission, Krakow, Poland; University of the National Education Commission, Krakow, Poland

## Abstract

**Purpose:**

Physical activity has been widely recognized as crucial for promoting optimal physical health, mental well-being, and social functioning. Given its significant impact on overall quality of life, it is essential to identify factors that may hinder engagement in regular physical activity. This study aims to explore the relationship between emotional self-regulation, general procrastination, physical activity procrastination, and physical activity levels among adults. Understanding these mechanisms can inform the development of interventions aimed at increasing physical activity engagement and ultimately promoting overall health and well-being.

**Methods:**

186 adults from general population participated in an online survey. They completed questionnaires assessing emotional self-regulation, general procrastination, and physical activity procrastination. Physical activity level was measured based on self-reported average weekly exercise minutes over the past year. Participants were then categorized into four activity levels according to the World Health Organization’s guidelines for physical activity and sedentary behavior.

**Results:**

Our results showed that lower levels of emotional self-regulation were significantly associated with lower levels of physical activity. Furthermore, this relationship was serially mediated by both general procrastination and physical activity procrastination. Specifically, individuals with lower levels of emotional self-regulation had a greater tendency to engage in general procrastination, which subsequently increased their tendency to delay or avoid physically active behaviors. As a result, these individuals had lower overall engagement in regular exercise or training.

**Conclusions:**

The results suggest that emotional self-regulation plays a critical role in influencing procrastination and physical activity. Lower levels of self-regulation affect this indirectly through increased tendencies toward both general and specific form of procrastination related to engaging in physically active behaviors. These findings highlight the importance of targeting interventions aimed at improving emotional self-regulation skills to reduce procrastination and increase physical activity, thereby promoting healthier lifestyles and well-being. Future research should explore potential strategies to effectively address these barriers while promoting long-term adherence to physically active behaviors.

